# Identification of miR-4644 as a suitable endogenous normalizer for circulating miRNA quantification in hepatocellular carcinoma

**DOI:** 10.7150/jca.48903

**Published:** 2020-10-17

**Authors:** Jun Zhao, Xin-Chao Zhu, Xiao-Song Wu, Lin Wang, Can-Can Zhu, Ke Yang, Guo-Qing Deng, An Wang, Yong Liu, Wei-Dong Jia, Ling Zhu

**Affiliations:** 1 Center of Engineering Technology Research for Biomedical Optical Instrument, Anhui Institute of Optics and Fine Mechanics, Hefei Institutes of Physical Science, Chinese Academy of Sciences, Hefei 230031, China.; 2 Science Island Branch of Graduate School, University of Science and Technology of China, Hefei 230026, China.; 3 Department of General Surgery, Anhui Provincial Hospital & the First Affiliated Hospital of USTC, Division of Life Science and Medicine, University of Science and Technology of China, Hefei 230001, China.

**Keywords:** circulating microRNA, endogenous control, normalization in RT-qPCR, hepatocellular carcinoma

## Abstract

**Background:** Circulating microRNAs (miRNAs) have proved to be promising biomarkers for early diagnosis and therapeutic monitoring in cancers. Particularly for hepatocellular carcinoma (HCC), detection of circulating miRNA biomarkers as a new diagnostic approach has been written into the latest Guidelines for Diagnosis and Treatment of Primary Liver Cancer in China (2019 edition). However, no general consensus on an ideal endogenous normalizer for circulating miRNAs quantification has been reached, so it will affect the accuracy of quantitative results. In this study, we aim to identify a stable endogenous normalizer for analyzing circulating miRNAs.

**Methods:** Candidate miRNAs were selected by screening dataset GSE104310, as well as data statistics and analysis. Five commonly reference genes were chosen for further comparison and verification. Then, the expression levels of these genes in serum were analyzed by quantitative reverse transcription PCR (RT-qPCR) among four groups, including patients diagnosed with HCC, chronic hepatitis B (CHB), liver cirrhosis, and healthy subjects. Furthermore, the stability of target genes was evaluated using geNorm, NormFinder, comparative ΔCq programs, and validated by database. We also explored the availability of the miRNA combination, and compared the performance difference between combination and individuals, as well as the selectivity of miRNA references in the combinations.

**Results:** 11 candidate miRNAs were obtained, and miR-4644 stood out among these miRNAs, and proved to be much more stable than other endogenous miRNAs. Further study showed that miR-4644 exhibited higher stability and expression abundance than other commonly miRNA reference controls. Finally, we discovered the combination of miR-4644 and miR-16 revealed high performance in stability when compared to miRNA individuals. Furthermore, the combination consisted of references with closer nature could give rise to amplification effects in stability.

**Conclusions:** Our findings demonstrated that miR-4644 is an ideal endogenous normalizer for circulating microRNA quantification in hepatocellular carcinoma. Besides, combining miR-4644 with miR-16 into a whole as a reference control would greatly improve the accuracy of quantification.

## Introduction

Hepatocellular carcinoma (HCC) is one of the most common malignancies that occurs in liver, and accounts for 75% to 85% of primary liver cancers 1. Presently, the morbidity and mortality of HCC are so high that more than half of all new and death cases in the world appeared in China 2, 3. Clinical researches have demonstrated the overall 5-year survival rate was approximately 18% if diagnosed with terminal liver cancer 4. However, for early-stage patients, the 5-year survival rate will exceed 70% as long as with proper treatment 5. Thus, early diagnosis and treatment are critical for improving the survival rate of patients. For a long time, serological test, imaging examination, and tissue biopsy were several commonly used detection methods for clinical diagnosis and screening of HCC. But, higher false negative rate 6, 7, lower sensitivity 8 and greater trauma 9 are bottlenecks that restrict the development of these technologies. Therefore, it is particularly important to establish an early-warning system with high-efficiency and precision for the prevention and control of HCC.

MicroRNAs (miRNAs) are a special class of short non-coding RNA that play an important part in developments and physiological activities, and also can act as carcinogenic or tumor suppressor regulators 10, 11. Recently, a growing number of evidence has suggested that circulating miRNAs were highly correlated with a variety of diseases, and changed dynamically with the development of diseases 12-14. Since 2008, the circulating miRNA was first reported to be a novel biomarker for solid tumors 15, researchers have demonstrated the anomalous variations of expression for circulating miRNAs have occurred in the early stages of a tumor, thus it can be used for risk early warning of tumors in high-risk groups 16, 17. Currently, the liquid biopsy-based on miRNA molecular markers with high stability, availability, and non-invasion has become an important approach for clinical diagnosis, screening and monitoring 18, 19. In other words, it makes it possible to diagnose cancer by a single tube of blood.

Quantitative reverse transcription-polymerase chain reaction (RT-qPCR) is one of the most sensitive methods for miRNA quantification. Generally, with the help of reference genes for data normalization, RT-qPCR could achieve precise quantification of miRNAs. Therefore, the accuracy of expression levels for target miRNAs is heavily relying on the stability of selected reference genes 20. Presently, endogenous miRNAs, RNU6 (U6), and exogenous miRNA spike-ins are several commonly reference controls that used for normalization of miRNA quantification. However, U6 degrades so easily that is fairly unstable in serum, and the research has shown that U6 is not an ideal reference gene for quantifying circulating miRNAs 21. Certainly for the use of exogenous miRNA spike-ins, they may not be able to monitor real level of expression for endogenous miRNAs in real time if the serum samples are taken from patients who have not fasted, so the accuracy of quantitative data for target miRNAs would be seriously affected. By comparison, none of these will happen when employ the endogenous miRNA as reference control. It has been proved to be relatively stable and difficult to degrade in the body fluids, thus, making it the proper reference control for quantification of circulating miRNAs 22, 23. Recently, there are numerous reports on the use of endogenous miRNA references for analyzing circulating miRNAs, such as miR-16 24, miR-24 25. However, there is still no general consensus until now on reference controls for the normalization of miRNAs quantification in cancers 22, 26, let alone for analyzing miRNAs in the serum of HCC. Particularly for current diagnosis of HCC, circulating miRNA detection as a new diagnostic approach has been written in the latest Guidelines for Diagnosis and Treatment of Primary Liver Cancer in China (2019 edition). Consequently, it is extremely urgent to find more appropriate endogenous references for accurate quantification of HCC miRNAs.

To our knowledge, there were a few reports on the identification of endogenous miRNAs in HCC, and diverse standpoints in previous researches were also held in scholars. For instance, Hu et al. identified miR-1288 in plasma, and considered it as a stable endogenous normalizer for quantifying miRNAs in liver patients 27. However, the so-called stable miR-1288 cannot be detected in the serum array of Lin et al 28, so they had to use synthetic cel-miR-67 for evaluating serum miRNA levels. Given the broad application prospects of miRNA biomarkers used in the field of tumor diagnosis in the future, it is essential to identify a reliable endogenous miRNA for quantitative normalization.

Therefore, in this study, we aim to identify a stable endogenous normalizer for analyzing circulating miRNAs in hepatocellular carcinoma. After integrated analysis on the GEO dataset and TargetScan Human 7.2 database, and validated through clinical samples, candidate miR-4644 was found with high expression stability in serum. Furthermore, several miRNA reference genes (miR-16, miR-24, miR-103, miR-191, and U6) were chosen for investigating the performance when compared to miR-4644. Our results suggested that the stability and reliability of miR-4644 were better than other references, and the combination of miR-4644 and miR-16 was recommended as the optimal normalizer for circulating miRNA quantification.

## Materials and Methods

### Patients and ethics statement

Peripheral blood samples of participants were obtained from four groups, including patients diagnosed with HCC, with chronic hepatitis B, with HBV-induced liver cirrhosis, as well as healthy subjects. All participants were enrolled without any treatments, the patients with any history of cancer were excluded other than HCC, and non-symptoms of liver disease were found in the healthy controls. Furthermore, the recruited participants of these four groups have been confirmed that met the eligibility criteria (Supplementary Table S1). This study had been approved by the Institutional Research Ethics Committee of the Anhui provincial hospital (Ethical No. 2019KY66), and all participants enrolled had signed the written informed consents.

### Study design

**Figure 1:**
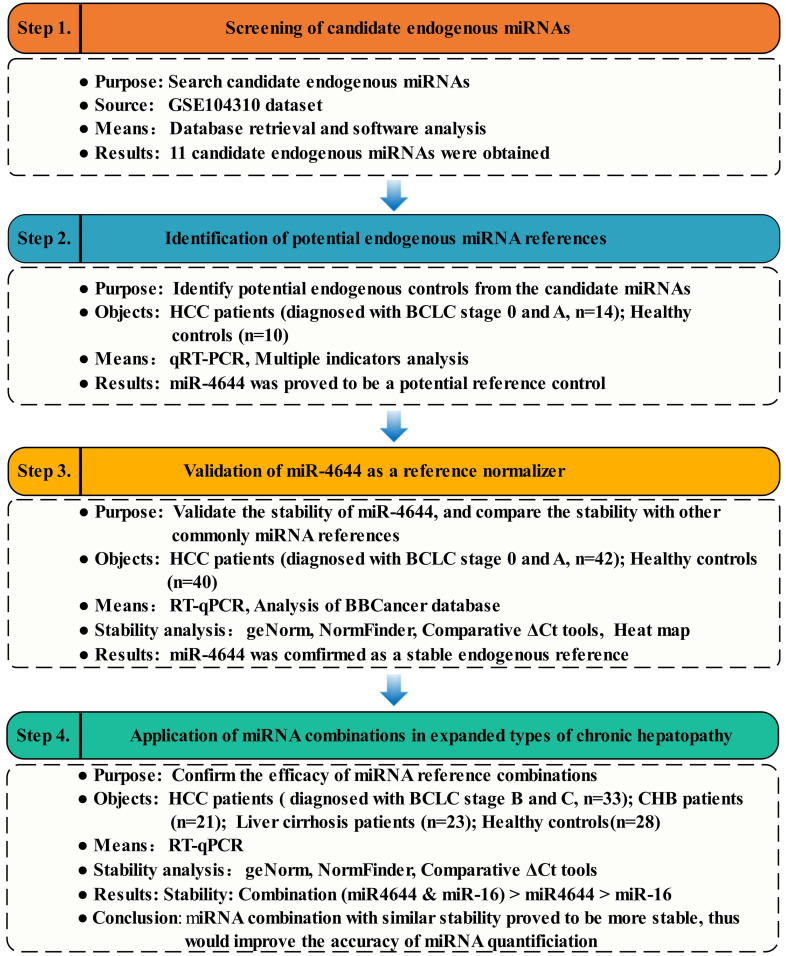
Workflow diagram illustrating strategy for identifying and evaluating endogenous miRNA normalizers.

Given that the genesis of HCC is resulted from abnormal expression of genes, and expression of genes are regulated by their target miRNAs 29. Thus, the initial research was carried out and started from key genes of Gene Expression Omnibus (GEO) dataset (https://www.ncbi.nlm.nih.gov/), in order to discover some miRNAs valuable for further in-depth study. Then, the preliminary miRNAs were obtained using TargetScan Human 7.2 database (http://www.targetscan.org/vert_72/) 30. Meanwhile, the candidate miRNAs from the subsets of genes were obtained by Venn diagram. Finally, the expression patterns of these candidate miRNAs in the serum samples were verified using RT-qPCR. To confirm the reliability and the stability of potential miRNA references, on one hand, the larger clinical sample size was applied. On the other hand, several commonly reference genes were tested and compared. For verification, the differential expression of these selected miRNAs were analyzed using blood-based biomarkers (BBCancer) database (http://bbcancer.renlab.org/) 31. Additionally, the comparison between a combination of references and its individuals was made for efficacy evaluation. In brief, the overview of strategy for identifying and evaluating endogenous miRNA normalizers was shown in **Figure 1.**

### Sample preparation and RNA extraction

For serum preparation, morning peripheral blood was drawn into vacuum tubes, and kept at room temperature at least 60 min, followed been centrifuged at 1000 × g for 10 min at 4 °C. Then, the supernatants were transferred to 2.0 mL sterile tubes, and stored at -80 °C for subsequent assays. The procedures of this pretreatment were carried out in accordance with workflow 32. Total RNA was separated and purified using TRIzol LS reagent and Phasemaker tubes complete system (Invitrogen, California, USA) according to the operation manual. In particular, RNA spike-in kit (QIAGEN, Hilden, Germany) was used for controlling the quality of RNA isolation. The concentrations and quality of pure RNA were measured by NanoDrop 2000 (Thermo Scientific, Wilmington, USA) and the RNA obtained with a value of 260/280 ≥ 1.80 would be used in subsequent experiments.

### RT-qPCR

For cDNA synthesis, a total of 50 ng total RNA per sample was transcribed to cDNA using Mir-X miRNA First-Strand Synthesis Kit (Clontech, California, USA), with a 10 µL reaction system for one hour at 37 °C, then, the reaction was terminated at 95°C for 1 min. The sequences of primers used throughout this study were shown in Table S2. Ultimately, the expression profile of cDNA was analyzed by qPCR using Mir-X miRNA RT-qPCR TB Green kit (Clontech, California, USA). The brief PCR protocol was as follows: denaturation at 95 °C for 10 s, then, conducted 45 cycles, and each cycle was about 5 s at 95 °C, as well as 20 s at 60 °C. Finally, a melting curve was conducted for analyzing the specificity of the amplified products. All the reactions involved were run on LightCycler 96 (Roche, Basel, Switzerland), and each data was repeated three times.

### Analysis of gene stability

Stability of candidate miRNAs expression among different samples was analyzed using three tools, such as geNorm 33, NormFinder 34, and comparative ΔCq method 35, they all using the respective algorithms to verify the stability of target genes. GeNorm was the tool to determine the most stable references by calculating an average expression stability coefficient (M value), the genes with high M value were gradually eliminated until the pair with the lowest M value was left, and the M value was recalculated for the remaining candidate genes after each high M value gene was excluded. Furthermore, geNorm can also determine the exact amount of reference controls utilizing analyzing pairwise variable coefficient (V value), that is when V_n_ / V_n+1_ < 0.15, the reference genes of n can satisfy the requirements of the experiment. NormFinder was a procedure based on analysis of variance for sorting the stability of genes, and it determined the more stable genes according to the lower M value, the principle was similar to the geNorm stated above. The comparative ΔCq method was an approach based on the total mean of Cq values in each given sample to normalize every miRNA data, then, the stability per miRNA was evaluated by the mean of standard deviations values of all samples. In particular, the stability varies inversely with the SD value. So the smaller the value, the better the stability; and this principle could be applied to other algorithms stated above, without exception.

### Statistical analysis and normalization

Student's *t-*test was used to evaluate the difference of miRNA expression between two groups (HCC vs healthy), and one-way ANOVA and nonparametric tests served for comparisons among four groups (HCC vs CHB vs liver cirrhosis vs healthy). Particularly, *p* value < 0.05 was considered statistically significant. To compare the expression difference of has-miR-4644, as well as other commonly reference genes, relative expression levels were measured by 2^-ΔCq^ between HCC and health groups, and the data were normalized to spiked *c. elegans* miR-67 (cel-miR-67). All statistical analyses and graphics were performed by GraphPad Prism version 7.04 (San Diego, CA, USA). Standard deviation (SD), interquartile range (IQR), and CV value were calculated for Cq values of those genes to evaluate the degree of dispersion between groups.

## Results

### Patient characteristics

In this study, a total of 211 subjects were included. Among of them, 89 patients diagnosed with HCC, 44 patients with chronic hepatopathy (21 individuals with chronic hepatitis B, and 23 individuals with HBV-induced liver cirrhosis) were gathered at general surgery department of Anhui Provincial Hospital (Hefei, China) between June 2019 and January 2020. Besides, 78 healthy controls were chosen from the hospital at the same period. The clinical characteristics of these participants recruited were presented in **Table 1.** For each stage of research, the age and gender of participants were considered fully before adopting.

### Screening of candidate miRNAs

Following the route of the design scheme stated above, the public dataset (GSE104310) was analyzed and the genes of CLEC4G and CLEC4M were found with a significant difference between tumors and controls (Figure S1). To our knowledge, recent studies have shown that this pair of genes is closely associated with the occurrence and progression of cancer, especially in HCC 36, 37. Hence, the study on miRNAs associated with the genes aroused our interests, and we expected to discover some targets more valuable for further research. Finally, 11 candidate miRNAs (Figure S2, Table S3) were obtained using TargetScan Human 7.2 database, as well as Venn diagram tool.

### Identification of potential endogenous miRNA references in human serum

**Figure 2:**
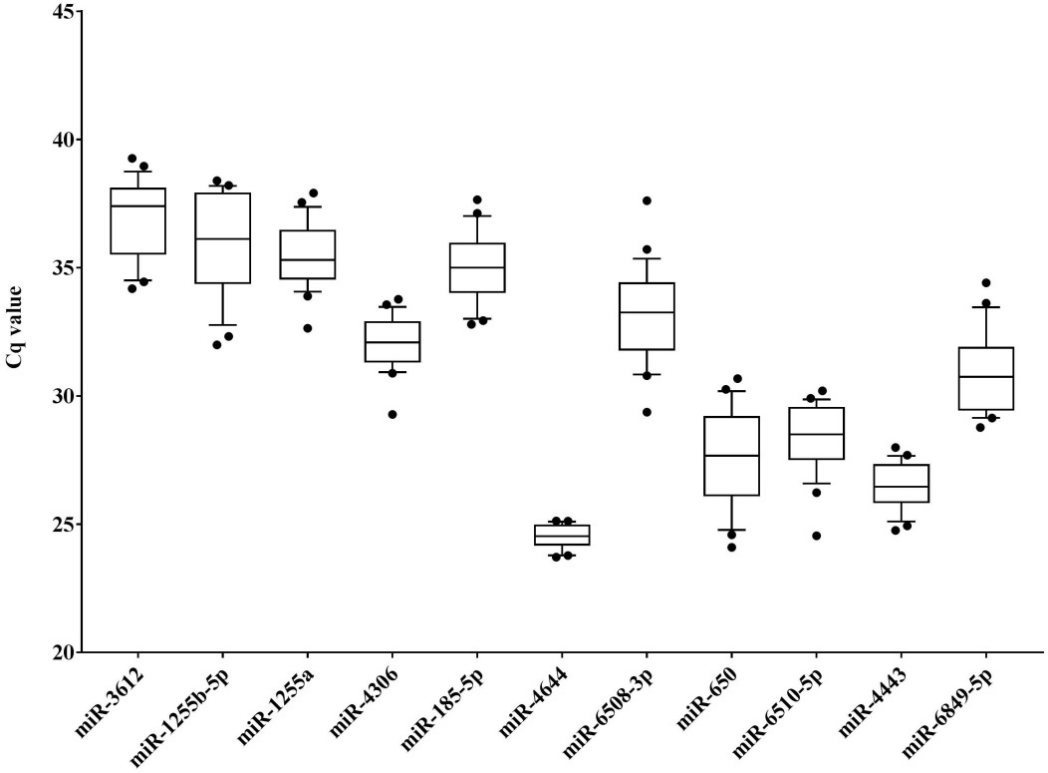
** Distribution of raw cycle (Cq) values of candidate miRNAs in serum samples (HCC: n = 14; healthy: n = 10).** Data are shown as box and whiskers plot (10th - 90th percentile), the dots represented outliers of Cq values.

In order to identify potential endogenous controls from the candidate miRNAs, the 11 target miRNAs (Table S3) in serum samples of HCC patients (diagnosed with BCLC stage 0 and A, n = 14) and healthy controls (n = 10) were tested using RT-qPCR approach, and the distributions of raw Cq values of these miRNAs was presented in **Figure 2.** Apparently, the Cq values of miR-4644 were more concentrated in total samples (n = 24), indicating that it might have high stability than other endogenous miRNAs. Meanwhile, multiple indicators, such as standard deviation (SD), interquartile range (IQR), and coefficient of variation (CV) value were also calculated for validating this consequence, and the results of data were shown in **Table 2.** It was not hard to find that the values of SD, IQR, and CV of miR-4644 were almost the lowest among these endogenous miRNAs; thus, we can conclude that miR-4644 was the most stable one of these candidate endogenous miRNAs. In addition, miR-4644 was also found with considerably lower Cq value than other miRNAs. In particular, Cq value varies inversely with gene expression abundance under the same conditions, namely the smaller the Cq value, the higher the expression abundance 38. Thus, indicating that miR-4644 was highly expressed in the serum. Without a doubt, high expression abundance was essential for an ideal reference gene. Given the above considerations, miR-4644 was proved to be much more stable than other endogenous miRNAs, and it was of great importance for further research if as a valuable endogenous reference.

### Validation of miR-4644 as a normalizer by RT-qPCR

**Figure 3:**
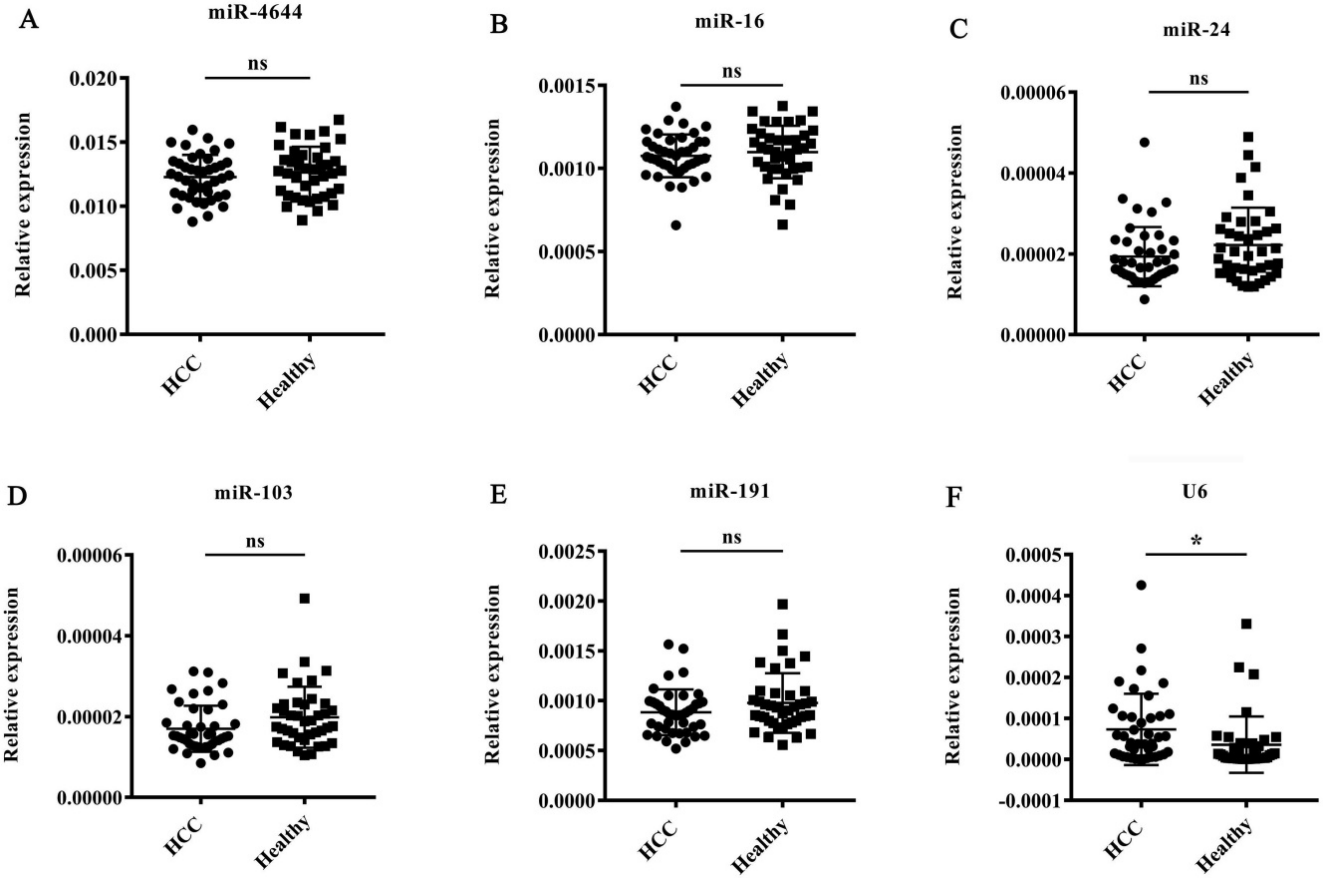
** Expression patterns of miR-4644, and other five known reference genes in HCC and health groups (HCC: n = 42; healthy: n = 40).** The expression levels were normalized to spiked cel-miR-67. **(A)** miR-4644, **(B)** miR-16, **(C)** miR-24, **(D)** miR-103, **(E)** miR-191, **(F)** U6. Data are shown as means ± SD, ns = not significant, * *p* < 0.05.

To validate the stability of miR-4644 as an endogenous normalizer for miRNA quantification in hepatocellular carcinoma, the expression patterns of miR-4644 were further analyzed by RT-qPCR in 42 HCC patients (diagnosed with BCLC stage 0 and A) and 40 healthy controls. In addition, four commonly reference genes, such as miR-16, miR-24, miR-103, and miR-191 reported in serum 22, 39, 40, as well as U6 snRNA provided in the quantitative kit were also used for validating at the same time. Finally, the expression levels of miR-4644, and those genes stated above were shown in **Figure 3.** There were no significant difference on miR-4644, as well as the other commonly miRNA references between HCC and healthy controls, except for the U6 gene. Besides, we have also found that the average raw Cq value of miR-4644 was even lower than these commonly reference controls. In other words, the miR-4644 might have a higher abundance of expression than these traditional reference controls. Hence, we deduced that miR-4644 has the potential to be an internal reference control due to its characteristics with excellent stability and expression abundance, and it was necessary for further confirmation.

### Stability analysis by software algorithms

**Figure 4:**
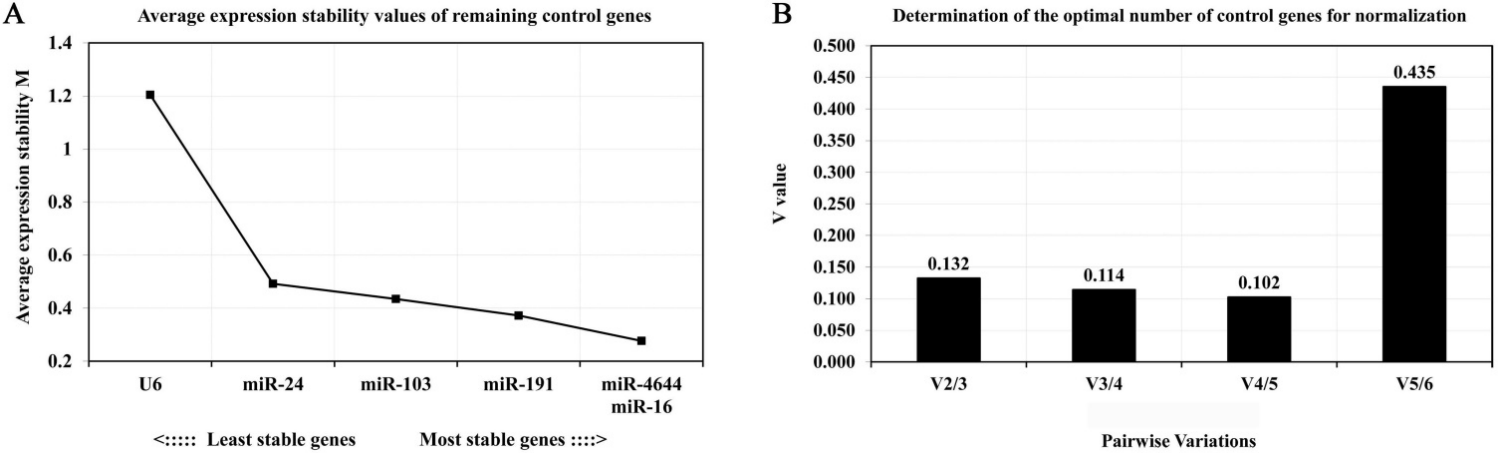
** Expression stability and the optional number of reference genes by geNorm analysis. (A)** Average values of expression stability for remaining controls. **(B)** Pairwise variation coefficient (V value) for determining the number of optimal references.

**Figure 5:**
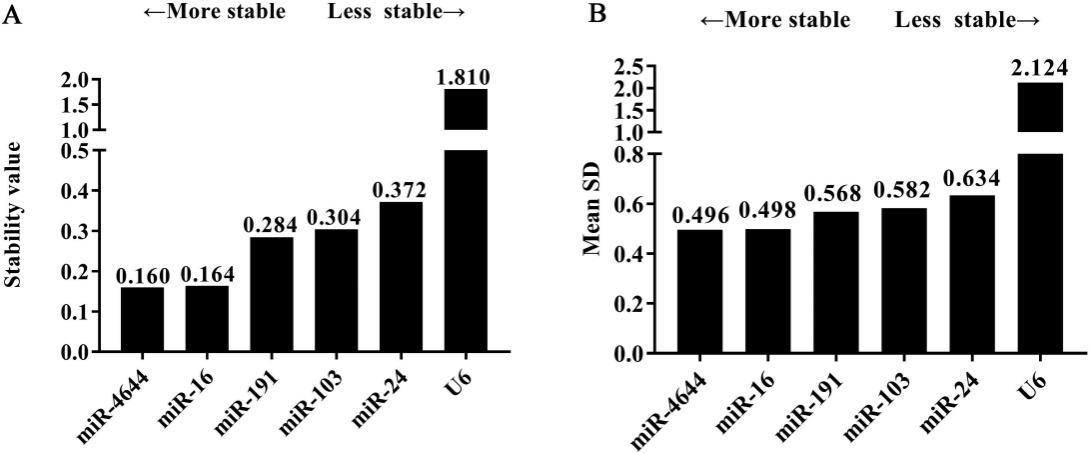
** The tendency of gene stability analyzed using software algorithms. (A)** NormFinder program. **(B)** Comparative ΔCq program.

In order to further confirm the stability of miR-4644, and compared its performance with other reference genes, geNorm, NormFinder, and comparative ΔCq programs were adopted for further verification. Finally, the trend chart on the stability of these genes using the geNorm algorithm was presented in **Figure 4A**, suggesting that miR-4644 was the most stable one among the selected genes. In addition, the stability and ranking of the target genes were shown in **Table 3.** In particular, the smaller the value of the target gene, the higher the stability, and vice versa 33. In this program, the expressions of miR-4644 and miR-16 showed higher stability on account of the lower M values, followed were miR-191, miR-103, and miR-24 (**Table 3**). Generally, the threshold value of M in geNorm is 1.5, and only when M value less than 1.5 can indicate that the gene with high stability. Here, the M values of all miRNAs were almost less than 1.5, except for U6. This might mean that it has poor stability on expression in serum. In addition, in terms of determining the optimal number of references, the results of V_2_ / V_3_, V_3_ / V_4_, and V_4_ / V_5_ were all less than 0.15 (**Figure 4B**), suggesting that it was sufficient to select two reference genes for great performance. Hence, both of miR-4644 and miR-16 were the most appropriate choice, and combined them into a whole might better enhance the accuracy of quantitative results. For NormFinder analysis, miR-4644 was also recognized as the most stable reference due to the lowest M value at 0.160 (**Figure 5A**), and the ranking of other genes was consistent with previous analyses using geNorm (**Table 3**). Finally, the stability of these genes was deeply verified by the comparative ΔCq procedure. In this analysis, we first calculated the ΔCq values (target gene Cq - total mean Cq) of each sample, then, the standard deviations of the target gene in all selected samples were obtained for evaluating the stability. Here, the trends of stability presented in mean SD were shown in **Figure 5B**, indicating that miR-4644 remained to be the most stable gene (mean SD = 0.496) in all reference genes. More importantly, the ranking of stability using this method was the same as those of the previous two analyses (**Table 3**). Consequently, based on the above findings, we can draw the conclusion that miR-4644 was more stable than the other commonly reference genes, and it can be regarded as a suitable miRNA reference control. Besides, it needs to be stressed that the expression of U6 fluctuated greatly in serum, thus we insisted that it was completely incapable of circulating miRNA analysis owing to this poor stability.

### Verification of stability using the public database

To further validate the stability of expression for miR-4644 and selected miRNA references, a public database, namely BBCancer was analyzed and the similar findings and conclusions stated above were obtained in this database. Figure S3 described the differential expression analyses of blood miRNAs in 13 cancers types. We found that the expression difference of miR-4644 in 13 blood of cancers types was significantly lower other commonly miRNA references when relative to the normal tissue. Apparently, the same results could be presented more visually in heat map (Figure S4). Therefore, it can be concluded that the stability of miR-4644 was higher than that of the commonly miRNA references. Furthermore, miR-4644 was probably an ideal housekeeping gene by reason of its higher stability of expression between tissue and blood.

### Application of miRNA combinations in expanded types of chronic hepatopathy

**Figure 6:**
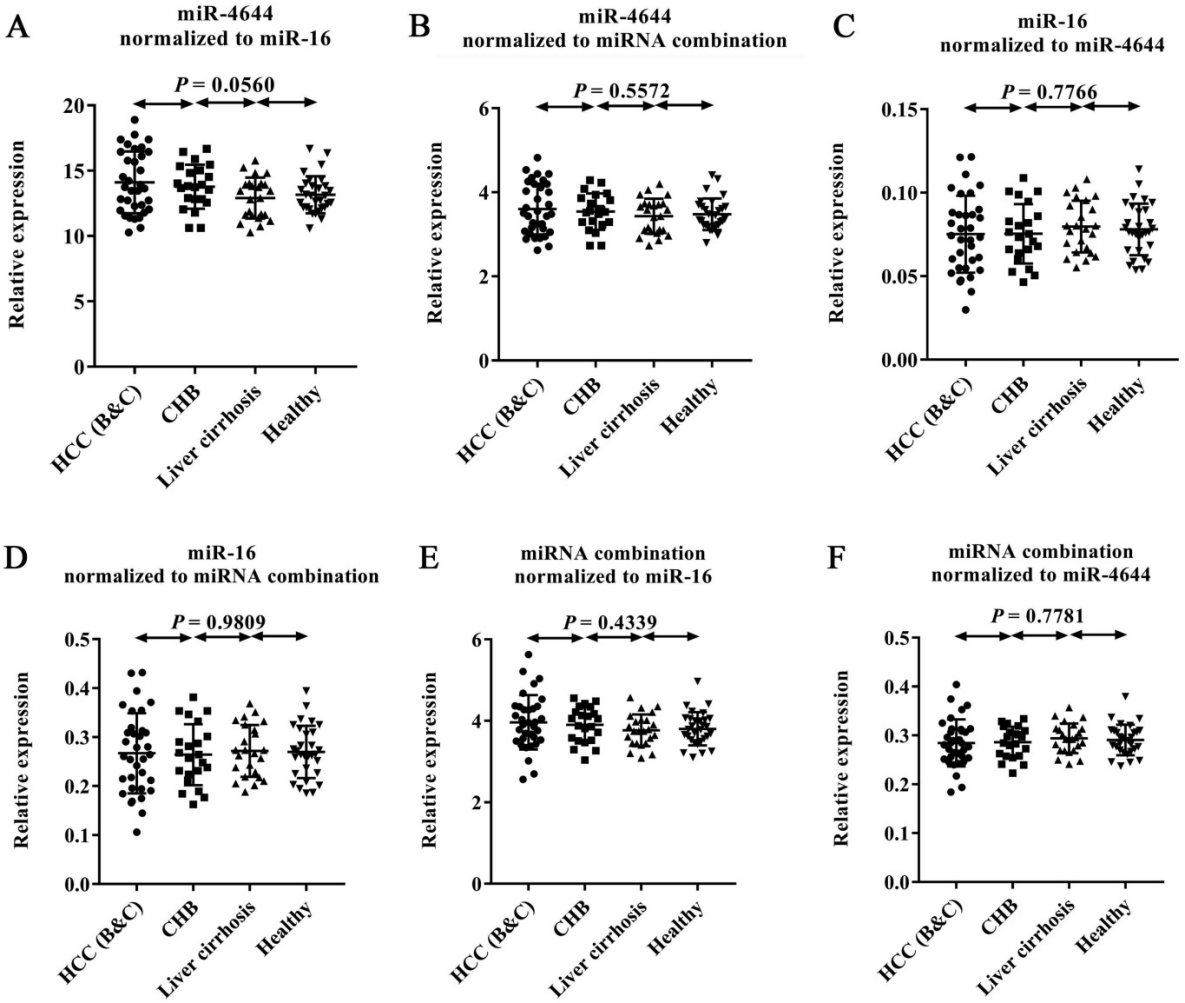
** Expression levels of endogenous miRNAs and their combinations by RT-qPCR. The relative expression levels of each gene was normalized to other two references between HCC patients (stage B or C, n = 33), CHB (n = 21), liver cirrhosis (n = 23), and healthy controls (n = 28). (A)** miR-4644 normalized to miR-16. **(B)** miR-4644 normalized to their combination. **(C)** miR-16 normalized to miR-4644. **(D)** miR-16 normalized to miRNA combination. **(E)** miRNA combination normalized to miR-16. **(F)** miRNA combination normalized to miR-4644. Data are shown as means ± SD.

In order to confirm the usability of miRNA combination as a reference control for quantification of circulating miRNAs, we combined miR-4644 and miR-16 into a whole for further validation. In this stage, 105 additional donors, including 33 patients with diagnosed HCC (confirmed with BCLC stage B or C), 21 patients with CHB, 23 patients with HBV-induced liver cirrhosis, and 28 healthy controls were involved into the test. The expression levels of miR-4644, miR-16, and their combination across the above four types were illustrated in **Figure 6.** In particular, for comparing the performance differences among them when as the reference control, the relative expression level of each one was normalized to the other two references. Finally, no significant difference (*p* > 0.05) among four groups was found in miR-4644, miR-16, and their combination, when normalized to any two of them. Thus, suggesting that both of miR-4644 and miR-16 have high stability of expression in expanded types of chronic hepatopathy. Furthermore, the relative expression difference of miR-4644, miR-16, and their combination could be distinguished from *p* value calculated by one-way ANOVA and Nonparametric tests. On the one hand, the *P* value of individuals (such as miR-4644, shown in **Figure 6B**) normalized to the combination was greater than it normalized to the miR-16 (**Figure 6A**) among the four subjects. Similarly, the same was true for the miR-16 (**Figure 6D, 6C**). Thus, it can be suggested that the performance of the combination was better than any one of the individuals if treated as the reference control. In other words, the accuracy of quantitative results would be improved if combined reference individuals into a whole. On the other hand, the *p* value of combination normalized to the miR-4644 (**Figure 6F**) was greater than it normalized to the miR-16 (**Figure 6E**); this proved again that the miR-4644 was superior to miR-16 when as the reference control. In summary, the combination consisted of miR-4644 and miR-16 was demonstrated to be a suitable reference control, and it would contribute to the achievement of better effect for miRNAs analyses.

**Figure 7:**
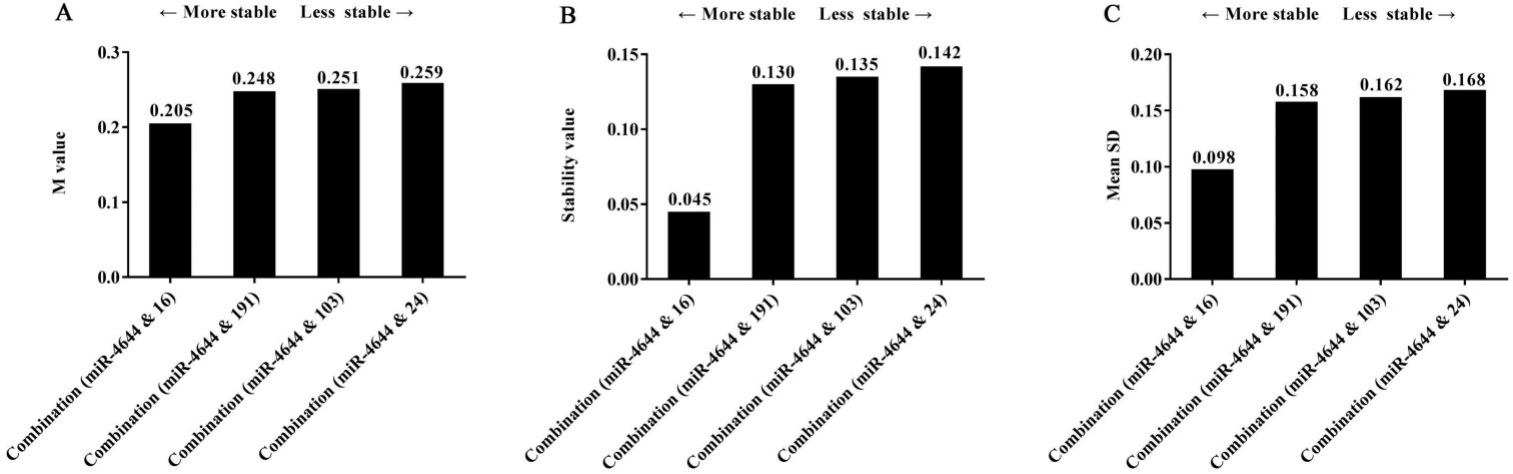
** The stability of four combinations analyzed using different algorithms. (A)** geNorm program. **(B)** NormFinder program. **(C)** Comparative ΔCq program.

Frankly speaking, while we have seen the remarkable performance of miRNA combination as a suitable reference control, combined which individuals into a whole could receive better results were not clear. Hence, in order to explore this doubt, several miRNA references used above were made into combinations; the stability of these combinations was also evaluated by geNorm, NormFinder, and comparative ΔCq tools. And the comparisons of stability in four combinations selected were shown in **Figure 7.** Firstly, the same results were achieved for the ranking of four combinations when analyzed using different programs. Furthermore, the values of the combination of has-miR-4644 and has-miR-16 were all less than other combinations, indicating that the combination of has-miR-4644 and has-miR-16 that has similar stability was greater than that with a greater difference in stability. More importantly, we found that the values of miRNA combinations (miR-4644 & 191, miR-4644 & 103, miR-4644 & 24) with a greater difference have little difference in the stability (**Figure 7A-C**). However, the value of the combination (miR-4644 & 16) was significantly less than other three combinations (**Figure 7B, 7C**). Therefore, it was not hard to see that the combination of miRNA references with comparable stability might be more suitable for a reference control, and only in this way can greatly enhance the accuracy of miRNA quantification.

## Discussion

Currently, the liquid biopsy-based on miRNA biomarkers have been written in the Guidelines for Diagnosis and Treatment of Primary Liver Cancer in China (2019 edition), and the experts believed that the diagnostic protocol would play a vital part in early diagnosis and efficacy evaluation for HCC. Therefore, it is of profound significance for achieving accurate quantification of miRNAs for clinical applications in the future. However, there is still no general consensus until now on reliable endogenous references for normalization of miRNAs quantification 39. Particularly for endogenous miRNA control in HCC, there was a dispute among researchers. For instance, Hu et al. discovered and considered miR-1228 as a stable endogenous control for the quantification of plasma miRNAs in patients with hepatopathy 27. However, this so-called invariant miR-1228 was undetectable in an array assay of Lin et al. 28, and they had to use synthetic cel-miR-67 to analyze serum miRNA levels. To our knowledge, it was probably due to the different types of samples they tested. Without a doubt, there were few reports until now on the identification of miRNA reference in the serum of HCC, thus will seriously affect the accuracy of miRNA quantification in serum. Hence, it is particularly urgent to search for an optimal endogenous miRNA normalizer in the serum of HCC.

Hence, we aimed to identify a satisfactory endogenous miRNA reference for the quantification of circulating miRNAs in the serum of HCC. Frankly speaking, in this study, the new discovery of miR-4644 as a miRNA normalizer might be a certain amount of chance and luck. Initially, a pair of key genes, namely CLEC4G and CLEC4M were found with significant difference between tumors and controls, when retrieved a public dataset (GSE104310) of hepatocellular carcinoma. And to our knowledge, this pair of genes has been proved to be closely associated with the occurrence and progression of cancer, even in the HCC. Besides, the majority of genes were regulated by the miRNAs. Thus, the miRNAs associated with the genes aroused our interests, and we expected to discover some miRNAs valuable for further research. Finally, miR-4644 stood out from the candidate miRNAs, and was deemed as an ideal normalizer due to its less variability and high abundance in expression. Thus, it was well worthy of further study.

Afterwards, in order to evaluate and confirm the reliability of miR-4644 for a reference normalizer, two measures were adopted for this validation phase. On one hand, a total of 82 samples (HCC patients, n = 42; healthy controls, n = 40) were included in the assay for reducing the influence of certain chance factors on the effects of experiment. On the other hand, several commonly reference controls reported in the serum of other diseases were selected and tested in this study together 23, 41. We aim to compare the difference between miR-4644 and those known references in stability. Thus, the reliability and stability of miR-4644 as a reference normalizer were guaranteed. Finally, our results indicated that no significant difference was observed in the expression of these genes between HCC and health groups. Importantly, the raw Cq values of miR-4644 were also found to be lower than the conventional reference controls in the same circumstances, and generally the lower Cq values associated with the higher abundance 38. Hence, we can conclude that the expression abundance of miR-4644 was higher than other commonly reference controls. Without a doubt, the high expression abundance was important for an ideal reference gene 42. Presently, to our knowledge, miR-16 was one of the known reference controls with the highest expression abundance 43, 44, but at the moment, its advantage had lost in this respect, and might be replaced by miR-4644. To our knowledge, this was the first report that a new endogenous miRNA (miR-4644) has higher expression abundance than any other miRNAs.

Certainly, the stability of miR-4644, as well as other known references was evaluated using geNorm 33, NormFinder 34, and comparative ΔCq procedures 35. Thus, the performance of these reference genes could be confirmed in different algorithms. Unlike the previous studies on the identification of other miRNA normalizers, the ranking of miR-4644 was fairly stable, indicating that the reliability of the results was verified. Interestingly, geNorm tool suggested that optimal performance could be reached if combined two genes into a whole. To our knowledge, there was a precedent on the research of miRNA combination as promising biomarker, and it has been demonstrated that this combination possessed high diagnostic accuracy in HCC diagnosis 45. But that was not known yet for the combination of miRNA references. Hence, the study on performance of combination of miRNA references aroused our interests. In this study, miR-4644 and miR-16 were considered to be ranked in the top two in stable genes. So, this aroused our great interests in exploring this issue. In order to evaluate the stability on a combination of miRNAs (miR-4644 and miR-16), and expand its application in other types of chronic hepatopathy, another set of additional subjects, including patients diagnosed with stage B or C of HCC, with CHB, with liver cirrhosis, as well as healthy controls were chosen in this testing. In this phase of research, to compare the difference of performance between two references individuals and their combination as much as possible, we broke the conventional thinking mode, and firstly proposed a cross-normalization approach for analysis of reference genes. Namely, the relative expression level of each gene was normalized to any one of the other references in the group. Thus, the difference among them could be easily obtained through the *p* value using one-way ANOVA and nonparametric tests. Generally, the larger the *p* value, the less significant the difference, namely the stability was better. Without a doubt, the threshold value of *p* was 0.05, and the scheme was valid only if the value is greater than 0.05 for this project. In particular, the strategy might only apply to study on the difference of expression level for reference genes. Finally, the results were clear that the combination was demonstrated with great performance than miRNA individuals. Therefore, we can conclude that the combination of miR-4644 and miR-16 would be an ideal reference normalizer for circulating miRNA quantification. It should be noted that it was indeed a novel strategy for quantitative standardization when combined multiple references into a whole, thus can really improve the accuracy of quantification 46. However, a new question arose, namely which individuals were combined into a whole would give rise to amplification effect in their combinations. In order to explore the selectivity of miRNA references in the combinations, several miRNA individuals with different stability were combined separately for further study. The stability of these combinations was also validated using geNorm, NormFinder, and comparative ΔCq procedures, and it was not hard to see that the combination of miR-4644 and miR-16 was much more stable than other combinations. In briefly, these two miRNA references with closer stability could really give rise to an amplification effect in the respect of stability. In our opinion, the results suggested that an ideal reference combination should meet two essential demands: (1) the combination must consist of two reference genes which ranking the top in stability, (2) the stability is broadly similar across the individuals. Certainly, we guessed this conclusion might apply equally to reference controls of the other genes.

We acknowledge that there were still some limitations in this study. In terms of endogenous miRNAs normalizers in HCC, although our study has proved the miR-4644 was an ideal control in serum samples, its expression level and stability in the plasma sample were unknown. We emphasized this point because of miR-1280 as a reference control in plasma reported by Hu et al., but this was undetectable in an array of serum 27, 28, nevertheless, a new study has suggested that miR-1280 could be used as a serum normalizer in HCC 47. Furthermore, we have paid attention to the average Cq value of miR-1280 was greater than miR-4644 we identified. As the principle of the higher the value, the lower the abundance, hence, we can conclude that miR-4644 has higher abundance than miR-1280, and it might be more suitable for the normalizer in HCC.

## Conclusions

In this study, miR-4644 was demonstrated as a suitable endogenous miRNA reference with less expression variability and higher expression abundance in serum. Thus, it will contribute to accurate quantification of miRNA in HCC research. Meanwhile, further research has suggested that the reference combination of miR-4644 and miR-16 has fairly higher stability than each of them, and we also believed that a combination consisted of two reference individuals with similar characteristics in stability would give rise to amplification effect. In the long run, an increasing number of new miRNA normalizers will be identified, then the accuracy of miRNA quantification could be greatly improved if follow this principle.

## Supplementary Material

Supplementary figures and tables.

## Figures and Tables

**Table 1:** The clinical characteristics of participants recruited in this study

Clinical variable	Identification stage (step2, n=24)	Validation stage (step3, n=82)	Application stage(step3, n=105)
***HCC group, n***	14	42	33
**Age (years), n**			
< 60	11 (78.6%)	34 (81.0%)	15 (45.5%)
≥ 60	3 (21.4%)	8 (19.0%)	18 (54.5%)
**Gender, n**			
Female	4 (28.6%)	10 (23.8%)	8 (24.2%)
Male	10 (71.4%)	32 (76.2%)	25 (75.8%)
**ALT, n**			
Low (≤ 40 U/L)	9 (64.3%)	27 (64.3%)	22 (66.7%)
High (> 40 U/L)	5 (35.7%)	15 (35.7%)	11 (33.3%)
**AFP, n**			
Low (≤ 400 ng/mL)	10 (71.4%)	29 (69.0%)	7 (21.2%)
High (> 400 ng/mL)	4 (28.6%)	13 (31.0%)	26 (78.8%)
**Tumor size, n**			
Small (≤ 5 cm)	13 (92.9%)	38 (90.5%)	0
Large (> 5 cm)	0	0	33 (100%)
NA	1 (7.1%)	4 (9.5%)	0
***CHB group, n***			21
**Age (years), n**			
< 60			19 (90.5%)
≥ 60			2 (9.5%)
**Gender, n**			
Female			9 (42.9%)
Male			12 (57.1%)
**ALT, n**			
Low (≤ 40 U/L)			18 (85.7%)
High (> 40 U/L)			3 (14.3%)
**AFP, n**			
Low (≤ 400 ng/mL)			21 (100%)
High (> 400 ng/mL)			0
***Liver cirrhosis group, n***			23
**Age (years), n**			
< 60			18 (78.3%)
≥ 60			5 (21.7%)
**Gender, n**			
Female			7 (30.4%)
Male			16 (69.6%)
**ALT, n**			
Low (≤ 40 U/L)			20 (87.0%)
High (> 40 U/L)			3 (13%)
**AFP, n**			
Low (≤ 400 ng/mL)			23 (100%)
High (> 400 ng/mL)			0
***Healthy group, n***	10	40	28
**Age (years), n**			
< 60	9 (90%)	35 (87.5%)	26 (92.9%)
≥ 60	1 (10%)	5 (12.5%)	2 (7.1%)
**Gender, n**			
Female	4 (40%)	12 (30%)	9 (32.1%)
Male	6 (60%)	28 (70%)	19 (67.9%)
**ALT, n**			
Low (≤ 40 U/L)	10 (100%)	40 (100%)	28 (100%)
High (> 40 U/L)	0	0	0
**AFP, n**			
Low (≤ 400 ng/mL)	10 (100%)	40 (100%)	28 (100%)
High (> 400 ng/mL)	0	0	0

**Table 2:** Quantitative results of candidate endogenous miRNAs obtained by qRT-PCR

miRNA	Cq (Total, n=24)(Mean ± SD)	Cq (Total, n=24) IQR	Cq (Total, n=24) (CV: % Cq)
miR-3612	36.89 ± 1.50	2.47	4.05
miR-1255b-5p	35.85 ± 1.98	3.12	5.53
miR-1255a	35.50 ± 1.26	1.76	3.54
miR-4306	32.09 ± 1.05	1.47	3.28
miR-4644	24.51 ± 0.45	0.77	1.83
miR-185-5p	34.94 ± 1.32	1.79	3.77
miR-6508-3p	33.17 ± 1.80	2.19	5.43
miR-650	27.53 ± 1.91	2.93	6.92
miR-6510-5p	28.34 ± 1.35	1.85	4.76
miR-4443	26.47 ± 0.92	1.44	3.46
miR-6849-5p	30.86 ± 1.53	2.32	4.95

**Table 3:** The overall ranking according to the calculation of stability values using different analytical tools

Overall Ranking	Gene	geNorm		NormFinder		Comparative ΔCq	
		M value	Ranking	Stability value	Ranking	Mean SD	Ranking
1	miR-4644	0.857	1	0.160	1	0.496	1
2	miR-16	0.858	2	0.164	2	0.498	2
5	miR-24	0.977	5	0.372	5	0.634	5
4	miR-103	0.968	4	0.304	4	0.582	4
3	miR-191	0.946	3	0.284	3	0.568	3
6	U6	2.710	6	1.810	6	2.124	6
