# Supplementary material for: Identification of miR-4644 as a suitable endogenous normalizer for circulating miRNA quantification in hepatocellular carcinoma
Source: J Cancer. 2020 Oct 17;11(23):7032–44. doi: 10.7150/jca.48903 (PMC7592003; doi:10.7150/jca.48903)
Supplement: Supplementary file 1 — Supplementary figures and tables. [file jcav11p7032s1.pdf]

Identification of miR-4644 as a suitable endogenous normalizer for circulating miRNA quantification in hepatocellular carcinoma

Supplementary appendix

Table S1. Eligibility criteria for participants involved in study.

Table S2. The sequences of primers used in this study.

Table S3. Target endogenous miRNAs associated with mRNAs were selected with a score greater than 90.

Figure S1. Expression patterns of selected genes between primary tumors and paired non-tumors in hepatocellular carcinoma.

Figure S2. Venn diagram represents the miRNA co-communities between CLEC4G and CLEC4M.

Figure S3. Differential expression analyses of miRNAs in 13 cancers types.

Figure S4. Heatmap represents the differential expression of miRNAs in 13 cancers types.

Table S1. Eligibility criteria for recruited participants in this study.

Group	Criteria
Hepatocellular carcinoma	1. Diagnosed based on at least two imaging technologies (hepatic ultrasound together with CT and / or MRI)
	2. Confirmed by two experienced pathologists
	3. Patients were not treated with radiotherapy, chemotherapy, transarterial chemoembolization or ablation before collection of blood samples
Chronic hepatitis B†	1. HBsAg-positive > 6 months
	2. Serum HBV DNA > 20,000 IU/mL (10^5 copies/mL)
	3. Persistent or intermittent elevation in AST or ALT levels
Liver cirrhosis‡	1. With HBV infection
	2. If no biopsy available, diagnosis must be supported by two imaging technologies
	3. Confirmed by two experienced pathologists
Healthy control	1. Without liver disease, as well as other malignancy
	2. HBV serum markers (HBsAg, HBeAg, anti-HBe, and anti-HBc) were negative
	3. Normal AST and ALT levels

CT = Computed Tomography. MRI = Magnetic Resonance Imaging. HBV = hepatitis B virus. HBsAg = HBV surface antigen. HBeAg = HBV e antigen. anti-HBe = antibody against HBeAg. anti-HBc = HBV core antibody. AST = aspartate aminotransferase. ALT = alanine aminotransferase. † Refer to the American Association for the Study of Liver Diseases (AASLD) Practice Guidelines (2009). ‡ According to the description in Lancet (Schuppan D, Lancet 2008, 371: 838-51).

Table S2. The sequences of primers used in this study.

Genes	Primer Sequence (5′- 3′)
miR-3612	AACAAGAGGAGGCATCTTGAGAAAT
miR-1255b-5p	AACAAGCGGATGAGCAAAGAAAGT
miR-1255a	AACACGCAGGATGAGCAAAGAAA
miR-650	AACAAGACACTTGTTGGGATGACC
miR-6510-5p	AACAAGCAGCAGGGGAGAGAG
miR-4443	AACAAGTTGGAGGCGTGGGT
miR-6849-5p	AACAAGGAGTGGATAGGGGAGTG
miR-4306	AACACGCTGGAGAGAAAGGCA
miR-4644	AACACGCTGGAGAGAGAAAAGAGA
miR-185-5p	AACAAGTGGAGAGAAAGGCAGTTC
miR-6508-3p	AACAAGTGGGCCATGCATTTCTAG
miR-16	AACACGCTAGCAGCACGTAAATATT
miR-24	AACAAGTGGCTCAGTTCAGCAG
miR-103	AACAAGAGCAGCATTGTACAGG
miR-191	AACAAGCAACGGAATCCCAAAAG
cel-miR-67	CGGCTCACAACCTCCTAGAAAGAG
U6-F	GGAACGATACAGAGAAGATTAGC
U6-R	TGGAACGCTTCACGAATTTGCG

Table S3. Target endogenous miRNAs associated with mRNAs were selected with a score greater than 90.

**Target miRNAs**	**miRNAs to CLEC4M / CLEC4G Context++ Score Percentile**
miR-3612	99 / 99
miR-1255b-5p	99 / 99
miR-1255a	99 / 99
miR-650	99 / 99
miR-6510-5p	99 / 97
miR-4443	99 / 96
miR-6849-5p	99 / 94
miR-4306	97 / 94
miR-4644	97 / 94
miR-185-5p	97 / 93
miR-6508-3p	91 / 95

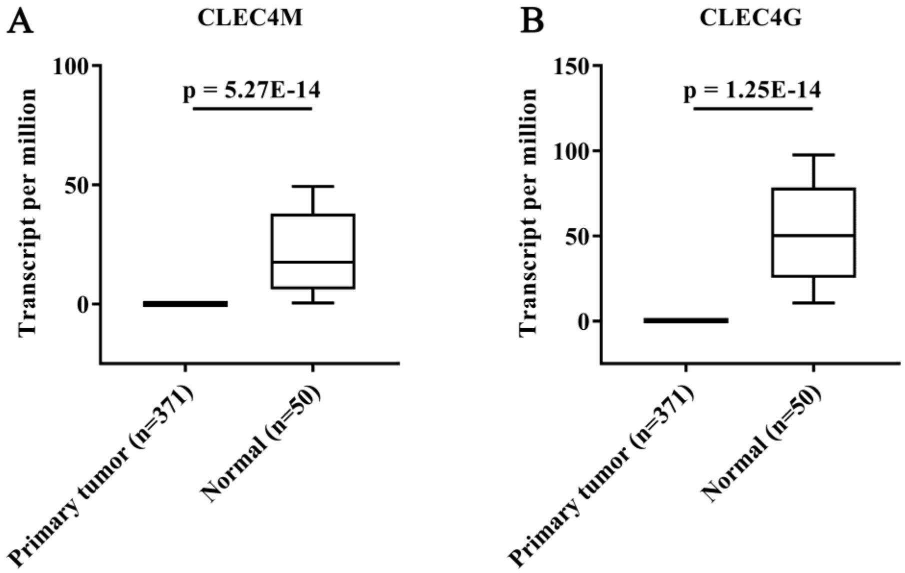

Figure S1. Expression patterns of selected genes between primary tumors and paired non-tumors in hepatocellular carcinoma. (**A**) CLEC4M and (**B**) CLEC4G were shown significant differences between primary tumor and normal tissue.

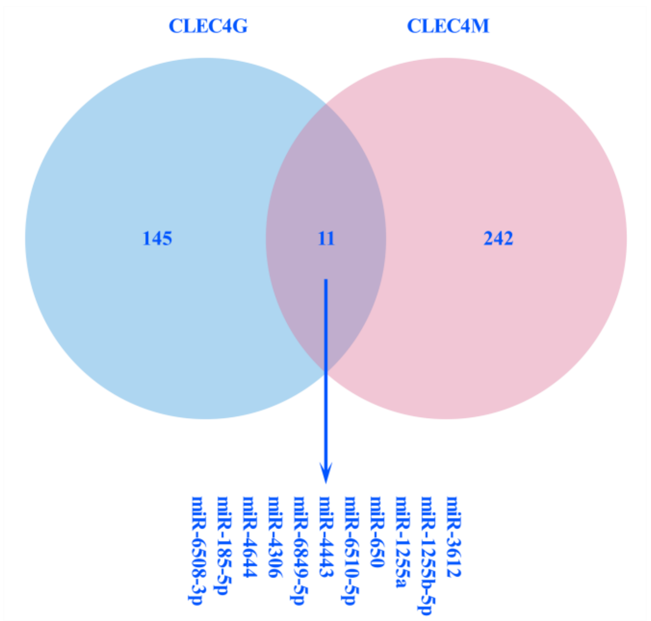

Figure S2. Venn diagram represents the miRNA co-communities between CLEC4G and CLEC4M.

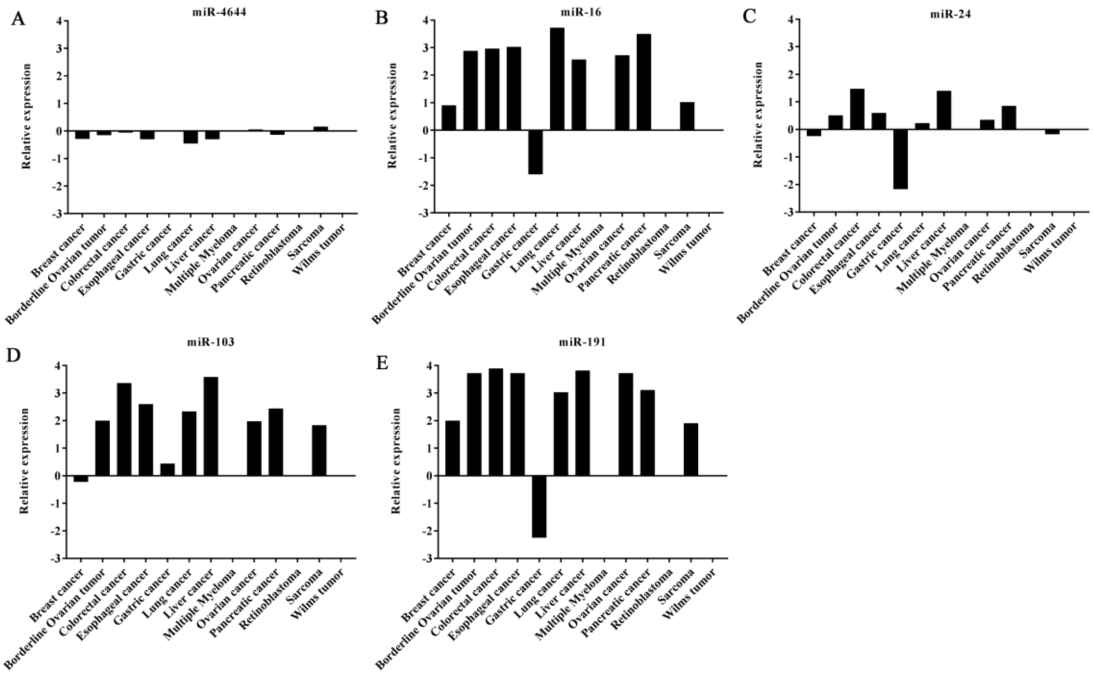

Figure S3. Differential expression analyses of miRNAs in 13 cancers types. The relative expression of each miRNA in blood was compared to its expression in normal tissue. (**A**) miR-4644, (**B**) miR-16, (**C**) miR-24, (**D**) miR-103, (**E**) miR-191.

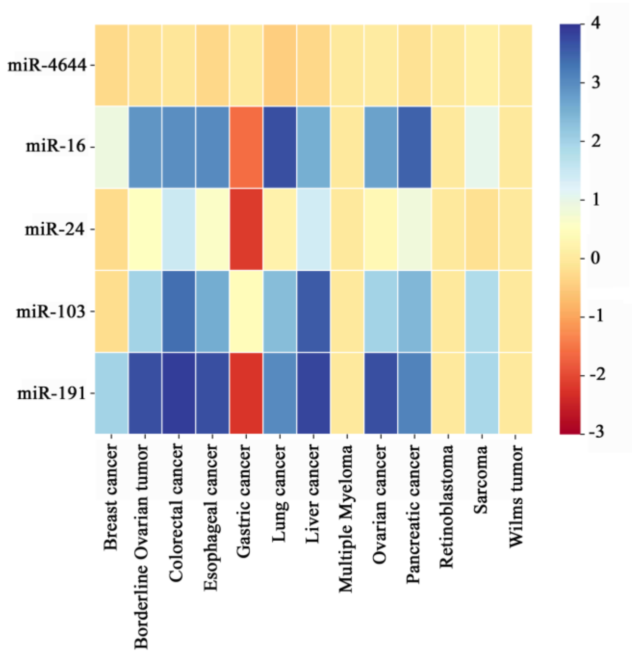

Figure S4. Heatmap represents the differential expression of miRNAs in 13 cancers types.
